# Hexaaqua­manganese(II) bis­[4-(pyridin-2-ylmeth­oxy)benzoate] dihydrate

**DOI:** 10.1107/S1600536811040943

**Published:** 2011-10-12

**Authors:** Li-Wei Zhang, Shan Gao, Seik Weng Ng

**Affiliations:** a Key Laboratory of Functional Inorganic Material Chemistry, Ministry of Education, Heilongjiang University, Harbin 150080, People’s Republic of China; b Department of Chemistry, University of Malaya, 50603 Kuala Lumpur, Malaysia; c Chemistry Department, Faculty of Science, King Abdulaziz University, PO Box 80203 Jeddah, Saudi Arabia

## Abstract

The Mn^II^ atom in the title salt, [Mn(H_2_O)_6_](C_13_H_10_NO_3_)_2_·2H_2_O, lies on a center of inversion in an octa­hedron of water mol­ecules. The cations, anions and uncoordinated water mol­ecules are linked by O—H⋯O and O—H⋯N hydrogen bonds into a three-dimensional network. The anion is essentially planar, with an r.m.s. deviation of all non-H atoms of 0.068 Å.

## Related literature

For the isotypic Co(II) salt, see: Zhang *et al.* (2011 ▶).
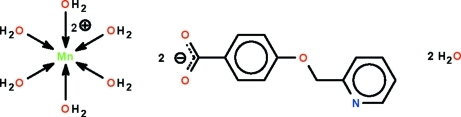


## Experimental

### 

#### Crystal data

[Mn(H_2_O)_6_](C_13_H_10_NO_3_)_2_·2H_2_O
*M* *_r_* = 655.51
Triclinic, 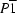

*a* = 7.4895 (18) Å
*b* = 7.6409 (18) Å
*c* = 13.791 (3) Å
α = 84.498 (4)°
β = 82.851 (5)°
γ = 72.576 (5)°
*V* = 745.7 (3) Å^3^
*Z* = 1
Mo *K*α radiation
μ = 0.51 mm^−1^
*T* = 293 K
0.19 × 0.12 × 0.11 mm

#### Data collection

Rigaku R-AXIS RAPID IP diffractometer
Absorption correction: multi-scan (*ABSCOR*; Higashi, 1995 ▶) *T*_min_ = 0.909, *T*_max_ = 0.946
7320 measured reflections
3387 independent reflections
1971 reflections with *I* > 2σ(*I*)
*R*_int_ = 0.053

#### Refinement

*R*[*F* ^2^ > 2σ(*F* ^2^)] = 0.060
*wR*(*F* ^2^) = 0.191
*S* = 1.07
3387 reflections
196 parameters
18 restraints
H-atom parameters constrained
Δρ_max_ = 0.62 e Å^−3^
Δρ_min_ = −0.70 e Å^−3^

### 

Data collection: *RAPID-AUTO* (Rigaku, 1998 ▶); cell refinement: *RAPID-AUTO*; data reduction: *CrystalClear* (Rigaku/MSC, 2002 ▶); program(s) used to solve structure: *SHELXS97* (Sheldrick, 2008 ▶); program(s) used to refine structure: *SHELXL97* (Sheldrick, 2008 ▶); molecular graphics: *X-SEED* (Barbour, 2001 ▶); software used to prepare material for publication: *publCIF* (Westrip, 2010 ▶).

## Supplementary Material

Crystal structure: contains datablock(s) global, I. DOI: 10.1107/S1600536811040943/bt5665sup1.cif

Structure factors: contains datablock(s) I. DOI: 10.1107/S1600536811040943/bt5665Isup2.hkl

Additional supplementary materials:  crystallographic information; 3D view; checkCIF report

## Figures and Tables

**Table 1:** Hydrogen-bond geometry (Å, °)

*D*—H⋯*A*	*D*—H	H⋯*A*	*D*⋯*A*	*D*—H⋯*A*
O1*w*—H11⋯O1	0.84	1.94	2.760 (3)	164
O1*w*—H12⋯O4*w*^i^	0.84	1.83	2.668 (4)	175
O2*w*—H21⋯O2	0.85	1.83	2.680 (3)	175
O2*w*—H22⋯O2^ii^	0.85	1.92	2.744 (3)	164
O3*w*—H31⋯O1^iii^	0.84	1.97	2.805 (3)	172
O3*w*—H32⋯N1^iv^	0.85	1.96	2.789 (4)	168
O4*w*—H41⋯O2	0.84	2.12	2.888 (4)	151
O4*w*—H42⋯O3*w*^v^	0.84	2.40	3.165 (4)	151

Symmetry codes: (i) 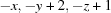; (ii) 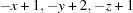; (iii) 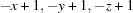; (iv) 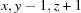; (v) 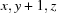.

**Fig. 1.**
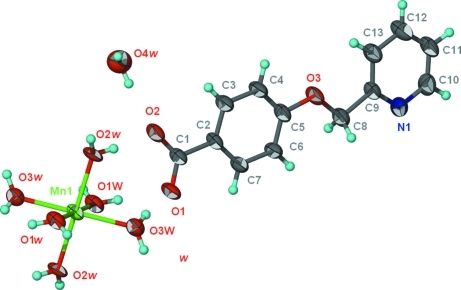
Anisotropic displacement ellipsoid plot (Barbour, 2001) of Mn(H2O)6.2(C13H10NO3).2H2O at the 50% probability level; hydrogen atoms are drawn as spheres of arbitrary radius.

## supplementary crystallographic information

### Comment

First-row transition metal dications form a plethora of metal dicarboxylates; however, occasionally, no direct metal–carboxylate bond is formed, and the product consists of hexaaquametal cations and carboxylate ions, the anion interacting indirectly in an outer-sphere type of coordination. 4-(Pyridin-2-ylmethoxy)benzoic acid is a commercially available carboxylic acid but there are no reports on its metal carboxylates. The reaction of the deprotonated acid with manganese(II) ions gives the hexaaquamanganese(II) salt (Scheme I, Fig. 1). The Mn^II^ atom in the salt lies on a center-of-inversion in an octahedron of water molecules. The metal atom interacts with the carboxylate ion indirectly, through the coordinated water molecules, in an outer-sphere type of coordination. The cations, anions and lattice water molecules are linked by O···H···O and O–H···N hydrogen bonds into a three-dimensional network (Table 1).

### Experimental

Manganese dichloride (1 mmol) was added to an aqueous solution of 4-(pyridin-2-ylmethoxy)benzoic acid (2 mmol) that was earlier been treated with 1*M* sodium hydroxide to a pH of 6. The filtered solution was set aside for several days, after which colorless prismatic crystals separated from solution.

### Refinement

Carbon-bound H-atoms were placed in calculated positions (C–H 0.93 Å) and were included in the refinement in the riding model approximation, with *U*(H) set to 1.2*U*(C). The water H-atoms were located in a difference Fourier map but were not refined. Their temperature factors were tied by a factor of 1.5 times.

The anisotropic temperature factors of the lattice water O were restrained to be nearly isotropic.

### Crystal data

**Table:**

[Mn(H_2_O)_6_](C_13_H_10_NO_3_)_2_·2H_2_O	*Z* = 1
*M**_r_* = 655.51	*F*(000) = 343
Triclinic, *P*1	*D*_x_ = 1.460 Mg m^−^^3^
Hall symbol: -P 1	Mo *K*α radiation, λ = 0.71073 Å
*a* = 7.4895 (18) Å	Cell parameters from 3613 reflections
*b* = 7.6409 (18) Å	θ = 3.1–27.5°
*c* = 13.791 (3) Å	µ = 0.51 mm^−^^1^
α = 84.498 (4)°	*T* = 293 K
β = 82.851 (5)°	Prism, colorless
γ = 72.576 (5)°	0.19 × 0.12 × 0.11 mm
*V* = 745.7 (3) Å^3^	

### Data collection

**Table:**

Rigaku R-AXIS RAPID IP diffractometer	3387 independent reflections
Radiation source: fine-focus sealed tube	1971 reflections with *I* > 2σ(*I*)
graphite	*R*_int_ = 0.053
ω scans	θ_max_ = 27.5°, θ_min_ = 3.1°
Absorption correction: multi-scan (*ABSCOR*; Higashi, 1995)	*h* = −9→8
*T*_min_ = 0.909, *T*_max_ = 0.946	*k* = −9→9
7320 measured reflections	*l* = −15→17

### Refinement

**Table:**

Refinement on *F*^2^	Primary atom site location: structure-invariant direct methods
Least-squares matrix: full	Secondary atom site location: difference Fourier map
*R*[*F*^2^ > 2σ(*F*^2^)] = 0.060	Hydrogen site location: inferred from neighbouring sites
*wR*(*F*^2^) = 0.191	H-atom parameters constrained
*S* = 1.07	*w* = 1/[σ^2^(*F*_o_^2^) + (0.0912*P*)^2^] where *P* = (*F*_o_^2^ + 2*F*_c_^2^)/3
3387 reflections	(Δ/σ)_max_ = 0.001
196 parameters	Δρ_max_ = 0.62 e Å^−^^3^
18 restraints	Δρ_min_ = −0.70 e Å^−^^3^

### Fractional atomic coordinates and isotropic or equivalent isotropic displacement parameters (Å^2^)

**Table:**

	*x*	*y*	*z*	*U*_iso_*/*U*_eq_	
Mn1	0.5000	0.5000	0.5000	0.0466 (3)	
O1	0.4142 (4)	0.8219 (3)	0.26773 (17)	0.0597 (7)	
O2	0.3600 (4)	1.0287 (3)	0.37937 (16)	0.0600 (7)	
O3	0.2466 (4)	1.5712 (3)	0.00213 (17)	0.0659 (8)	
O1w	0.2694 (3)	0.6085 (3)	0.41033 (17)	0.0540 (6)	
H11	0.2936	0.6767	0.3620	0.081*	
H12	0.1565	0.6540	0.4332	0.081*	
O2w	0.4579 (4)	0.7811 (3)	0.52917 (16)	0.0540 (7)	
H21	0.4325	0.8561	0.4797	0.081*	
H22	0.5051	0.8316	0.5677	0.081*	
O3w	0.3151 (4)	0.4446 (3)	0.63159 (16)	0.0571 (7)	
H31	0.3960	0.3577	0.6581	0.086*	
H32	0.2633	0.5227	0.6736	0.086*	
O4w	0.0862 (4)	1.2683 (4)	0.5115 (2)	0.0882 (10)	
H41	0.1300	1.1960	0.4663	0.132*	
H42	0.1734	1.2742	0.5427	0.132*	
N1	0.1726 (4)	1.7360 (4)	−0.2464 (2)	0.0554 (8)	
C1	0.3759 (5)	0.9845 (4)	0.2909 (2)	0.0471 (8)	
C2	0.3404 (5)	1.1379 (4)	0.2128 (2)	0.0453 (8)	
C3	0.3102 (5)	1.3176 (5)	0.2353 (2)	0.0520 (9)	
H3	0.3103	1.3441	0.2997	0.062*	
C4	0.2800 (6)	1.4580 (5)	0.1630 (2)	0.0562 (10)	
H4	0.2595	1.5785	0.1789	0.067*	
C5	0.2800 (5)	1.4203 (5)	0.0674 (3)	0.0510 (9)	
C6	0.3117 (5)	1.2423 (4)	0.0429 (2)	0.0512 (9)	
H6	0.3129	1.2168	−0.0218	0.061*	
C7	0.3420 (5)	1.1005 (5)	0.1161 (2)	0.0489 (9)	
H7	0.3635	0.9800	0.1000	0.059*	
C8	0.2382 (6)	1.5467 (5)	−0.0977 (2)	0.0548 (9)	
H8A	0.1441	1.4856	−0.1035	0.066*	
H8B	0.3590	1.4720	−0.1258	0.066*	
C9	0.1870 (5)	1.7363 (5)	−0.1506 (2)	0.0483 (8)	
C10	0.1244 (6)	1.8998 (6)	−0.2957 (3)	0.0645 (11)	
H10	0.1140	1.9023	−0.3623	0.077*	
C11	0.0898 (6)	2.0632 (5)	−0.2546 (3)	0.0640 (11)	
H11A	0.0563	2.1736	−0.2922	0.077*	
C12	0.1053 (7)	2.0615 (6)	−0.1567 (3)	0.0705 (12)	
H12A	0.0820	2.1706	−0.1261	0.085*	
C13	0.1566 (6)	1.8933 (5)	−0.1041 (3)	0.0584 (10)	
H13	0.1701	1.8879	−0.0377	0.070*	

### Atomic displacement parameters (Å^2^)

**Table:**

	*U*^11^	*U*^22^	*U*^33^	*U*^12^	*U*^13^	*U*^23^
Mn1	0.0650 (6)	0.0290 (4)	0.0490 (5)	−0.0172 (4)	−0.0134 (3)	0.0038 (3)
O1	0.095 (2)	0.0304 (13)	0.0560 (14)	−0.0190 (13)	−0.0203 (13)	0.0039 (10)
O2	0.101 (2)	0.0381 (13)	0.0470 (15)	−0.0260 (14)	−0.0228 (13)	0.0079 (10)
O3	0.117 (2)	0.0380 (14)	0.0449 (14)	−0.0237 (15)	−0.0232 (14)	0.0096 (10)
O1w	0.0678 (17)	0.0374 (13)	0.0593 (14)	−0.0172 (12)	−0.0174 (12)	0.0047 (10)
O2w	0.092 (2)	0.0215 (11)	0.0543 (14)	−0.0192 (12)	−0.0222 (13)	−0.0011 (9)
O3w	0.0719 (18)	0.0417 (14)	0.0531 (14)	−0.0116 (13)	−0.0064 (12)	0.0035 (11)
O4w	0.075 (2)	0.081 (2)	0.108 (2)	−0.0244 (18)	0.0048 (17)	−0.0131 (18)
N1	0.067 (2)	0.0510 (19)	0.0436 (17)	−0.0110 (16)	−0.0098 (14)	0.0072 (13)
C1	0.061 (2)	0.0328 (18)	0.051 (2)	−0.0166 (17)	−0.0157 (16)	0.0043 (14)
C2	0.058 (2)	0.0315 (16)	0.0491 (19)	−0.0164 (16)	−0.0146 (16)	0.0064 (13)
C3	0.079 (3)	0.0356 (18)	0.0445 (19)	−0.0180 (18)	−0.0180 (17)	0.0015 (14)
C4	0.088 (3)	0.0320 (18)	0.051 (2)	−0.0170 (19)	−0.0186 (19)	0.0015 (15)
C5	0.065 (2)	0.0371 (18)	0.051 (2)	−0.0164 (17)	−0.0152 (17)	0.0107 (15)
C6	0.077 (3)	0.0346 (18)	0.0427 (19)	−0.0147 (18)	−0.0149 (17)	−0.0009 (14)
C7	0.068 (2)	0.0324 (17)	0.049 (2)	−0.0159 (17)	−0.0134 (17)	0.0002 (14)
C8	0.070 (3)	0.042 (2)	0.053 (2)	−0.0189 (19)	−0.0115 (18)	0.0070 (16)
C9	0.055 (2)	0.0383 (19)	0.052 (2)	−0.0150 (17)	−0.0111 (16)	0.0062 (15)
C10	0.071 (3)	0.063 (3)	0.050 (2)	−0.008 (2)	−0.0110 (19)	0.0158 (19)
C11	0.082 (3)	0.041 (2)	0.068 (3)	−0.018 (2)	−0.020 (2)	0.0173 (18)
C12	0.096 (3)	0.044 (2)	0.078 (3)	−0.025 (2)	−0.031 (2)	0.0065 (19)
C13	0.086 (3)	0.046 (2)	0.048 (2)	−0.023 (2)	−0.0237 (19)	0.0065 (16)

### Geometric parameters (Å, °)

**Table:**

Mn1—O2w^i^	2.145 (2)	C2—C3	1.383 (5)
Mn1—O2w	2.145 (2)	C2—C7	1.388 (4)
Mn1—O1w	2.163 (2)	C3—C4	1.378 (4)
Mn1—O1w^i^	2.163 (2)	C3—H3	0.9300
Mn1—O3w	2.229 (2)	C4—C5	1.377 (5)
Mn1—O3w^i^	2.229 (2)	C4—H4	0.9300
O1—C1	1.252 (4)	C5—C6	1.377 (5)
O2—C1	1.279 (4)	C6—C7	1.394 (4)
O3—C5	1.374 (4)	C6—H6	0.9300
O3—C8	1.418 (4)	C7—H7	0.9300
O1w—H11	0.8420	C8—C9	1.521 (4)
O1w—H12	0.8447	C8—H8A	0.9700
O2w—H21	0.8498	C8—H8B	0.9700
O2w—H22	0.8503	C9—C13	1.360 (5)
O3w—H31	0.8419	C10—C11	1.361 (6)
O3w—H32	0.8451	C10—H10	0.9300
O4w—H41	0.8414	C11—C12	1.368 (5)
O4w—H42	0.8401	C11—H11A	0.9300
N1—C10	1.337 (4)	C12—C13	1.385 (5)
N1—C9	1.339 (4)	C12—H12A	0.9300
C1—C2	1.499 (4)	C13—H13	0.9300
			
O2w^i^—Mn1—O2w	180.00 (11)	C2—C3—H3	119.7
O2w^i^—Mn1—O1w	94.67 (8)	C5—C4—C3	120.2 (3)
O2w—Mn1—O1w	85.33 (8)	C5—C4—H4	119.9
O2w^i^—Mn1—O1w^i^	85.33 (8)	C3—C4—H4	119.9
O2w—Mn1—O1w^i^	94.67 (8)	O3—C5—C4	114.9 (3)
O1w—Mn1—O1w^i^	180.0	O3—C5—C6	124.7 (3)
O2w^i^—Mn1—O3w	85.49 (9)	C4—C5—C6	120.4 (3)
O2w—Mn1—O3w	94.51 (9)	C5—C6—C7	119.3 (3)
O1w—Mn1—O3w	93.70 (9)	C5—C6—H6	120.3
O1w^i^—Mn1—O3w	86.30 (9)	C7—C6—H6	120.3
O2w^i^—Mn1—O3w^i^	94.51 (9)	C2—C7—C6	120.5 (3)
O2w—Mn1—O3w^i^	85.49 (9)	C2—C7—H7	119.7
O1w—Mn1—O3w^i^	86.30 (9)	C6—C7—H7	119.7
O1w^i^—Mn1—O3w^i^	93.70 (9)	O3—C8—C9	107.4 (3)
O3w—Mn1—O3w^i^	180.0	O3—C8—H8A	110.2
C5—O3—C8	119.0 (3)	C9—C8—H8A	110.2
Mn1—O1w—H11	113.3	O3—C8—H8B	110.2
Mn1—O1w—H12	123.8	C9—C8—H8B	110.2
H11—O1w—H12	108.4	H8A—C8—H8B	108.5
Mn1—O2w—H21	113.9	N1—C9—C13	122.8 (3)
Mn1—O2w—H22	133.0	N1—C9—C8	114.5 (3)
H21—O2w—H22	106.7	C13—C9—C8	122.7 (3)
Mn1—O3w—H31	97.6	N1—C10—C11	124.1 (4)
Mn1—O3w—H32	123.2	N1—C10—H10	117.9
H31—O3w—H32	108.4	C11—C10—H10	117.9
H41—O4w—H42	110.0	C10—C11—C12	118.5 (3)
C10—N1—C9	116.7 (3)	C10—C11—H11A	120.7
O1—C1—O2	123.2 (3)	C12—C11—H11A	120.7
O1—C1—C2	119.5 (3)	C11—C12—C13	118.4 (4)
O2—C1—C2	117.2 (3)	C11—C12—H12A	120.8
C3—C2—C7	119.0 (3)	C13—C12—H12A	120.8
C3—C2—C1	120.8 (3)	C9—C13—C12	119.4 (4)
C7—C2—C1	120.2 (3)	C9—C13—H13	120.3
C4—C3—C2	120.6 (3)	C12—C13—H13	120.3
C4—C3—H3	119.7		
			
O1—C1—C2—C3	−175.7 (3)	C1—C2—C7—C6	−179.1 (3)
O2—C1—C2—C3	6.2 (5)	C5—C6—C7—C2	0.0 (6)
O1—C1—C2—C7	2.7 (5)	C5—O3—C8—C9	176.3 (3)
O2—C1—C2—C7	−175.4 (3)	C10—N1—C9—C13	−0.5 (6)
C7—C2—C3—C4	0.8 (6)	C10—N1—C9—C8	178.8 (3)
C1—C2—C3—C4	179.2 (4)	O3—C8—C9—N1	−179.6 (3)
C2—C3—C4—C5	−0.2 (6)	O3—C8—C9—C13	−0.3 (5)
C8—O3—C5—C4	−178.1 (3)	C9—N1—C10—C11	−0.1 (6)
C8—O3—C5—C6	1.8 (6)	N1—C10—C11—C12	0.2 (7)
C3—C4—C5—O3	179.4 (4)	C10—C11—C12—C13	0.3 (6)
C3—C4—C5—C6	−0.5 (6)	N1—C9—C13—C12	1.1 (6)
O3—C5—C6—C7	−179.3 (4)	C8—C9—C13—C12	−178.2 (4)
C4—C5—C6—C7	0.6 (6)	C11—C12—C13—C9	−1.0 (7)
C3—C2—C7—C6	−0.7 (6)		

Symmetry codes: (i) −*x*+1, −*y*+1, −*z*+1.

### Hydrogen-bond geometry (Å, °)

**Table:**

*D*—H···*A*	*D*—H	H···*A*	*D*···*A*	*D*—H···*A*
O1w—H11···O1	0.84	1.94	2.760 (3)	164.
O1w—H12···O4w^ii^	0.84	1.83	2.668 (4)	175.
O2w—H21···O2	0.85	1.83	2.680 (3)	175.
O2w—H22···O2^iii^	0.85	1.92	2.744 (3)	164.
O3w—H31···O1^i^	0.84	1.97	2.805 (3)	172.
O3w—H32···N1^iv^	0.85	1.96	2.789 (4)	168.
O4w—H41···O2	0.84	2.12	2.888 (4)	151.
O4w—H42···O3w^v^	0.84	2.40	3.165 (4)	151.

Symmetry codes: (ii) −*x*, −*y*+2, −*z*+1; (iii) −*x*+1, −*y*+2, −*z*+1; (i) −*x*+1, −*y*+1, −*z*+1; (iv) *x*, *y*−1, *z*+1; (v) *x*, *y*+1, *z*.
